# Combined 3D and hypoxic culture improves cartilage-specific gene expression in human chondrocytes

**DOI:** 10.3109/17453674.2011.566135

**Published:** 2011-04-05

**Authors:** Casper B Foldager, Anna B Nielsen, Samir Munir, Michael Ulrich-Vinther, Kjeld Søballe, Cody Bünger, Martin Lind

**Affiliations:** ^1^Orthopaedic Research Laboratory; ^2^Sports Trauma Clinic, Aarhus University Hospital, Aarhus, Denmark

## Abstract

**Background and purpose:**

In vitro expansion of autologous chondrocytes is an essential part of many clinically used cartilage repair treatments. Native chondrocytes reside in a 3-dimensional (3D) network and are exposed to low levels of oxygen. We compared monolayer culture to combined 3D and hypoxic culture using quantitative gene expression analysis.

**Methods:**

Cartilage biopsies were collected from the intercondylar groove in the distal femur from 12 patients with healthy cartilage. Cells were used for either monolayer or scaffold culture. The scaffolds were clinically available MPEG-PLGA scaffolds (ASEED). After harvesting of cells for baseline investigation, the remainder was divided into 3 groups for incubation in conditions of normoxia (21% oxygen), hypoxia (5% oxygen), or severe hypoxia (1% oxygen). RNA extractions were performed 1, 2, and 6 days after the baseline time point, respectively. Quantitative RT-PCR was performed using assays for RNA encoding collagen types 1 and 2, aggrecan, sox9, ankyrin repeat domain-37, and glyceraldehyde-3-phosphate dehydrogenase relative to 2 hypoxia-stable housekeeping genes.

**Results:**

Sox9, aggrecan, and collagen type 2 RNA expression increased with reduced oxygen. On day 6, the expression of collagen type 2 and aggrecan RNA was higher in 3D culture than in monolayer culture.

**Interpretation:**

Our findings suggest that there was a combined positive effect of 3D culture and hypoxia on cartilage-specific gene expression. The positive effects of 3D culture alone were not detected until day 6, suggesting that seeding of chondrocytes onto a scaffold for matrix-assisted chondrocyte implantation should be performed earlier than 2 days before implantation.

Native chondrocytes reside in a 3-dimensional (3D) network of extracellular matrix mainly consisting of collagen type 2 and glycosaminoglycans—primarily aggrecan. The avascular nature of cartilage results in low oxygen tensions. It has been proposed that chondrocytes in the deepest layers of the cartilage are exposed to oxygen tensions of less than 1% (Silver 1975). Culture of chondrocytes in 3D networks such as synthetic biodegradable scaffolds has been shown to be beneficial in terms of retained phenotype in prolonged culture (Takahashi et al. 2007), increased chondrogenic gene expression of dedifferentiated chondrocytes (Barlic et al. 2008), and enhanced proliferation (Lin et al. 2009) compared to monolayer culture. Similarly, hypoxic culture of chondrocytes has been shown to have several substantial effects on cell metabolism. Low oxygen tension induces chondrocyte-specific expression in mesenchymal and embryonic stem cells (Robins et al. 2005, Koay and Athanasiou 2008), stimulates redifferentiation of dedifferentiated and in vitro expanded chondrocytes (Domm et al. 2002, Malda et al. 2004, Murphy and Polak 2004, Martinez et al. 2008), and enhances matrix synthesis after prolonged exposure (Clark et al. 1991, Murphy and Sambanis 2001a, b, Coyle et al. 2009). The effects of hypoxia on chondrocytes are mediated through both sox9-dependent and sox9-independent pathways (Lafont et al. 2008). Although there is a general agreement about several positive effects of hypoxia on chondrocytes, negative effects in bioreactor culture has been described (Obradovic et al. 1999).

In treatment of cartilage, both autologous chondrocyte implantation (ACI) and its third-generation counterpart matrix-assisted chondrocyte implantation (MACI) require culture of autologous chondrocytes before reimplantation in the patient (Brittberg et al. 1994). Compared to the native environment, 2 specific factors are altered when chondrocytes are incubated in monolayer culture. The complex 3D structure of cartilage is replaced by a 2-dimensional surface. Secondly, chondrocytes are suddenly exposed to high levels of oxygen. Such culture conditions could alter the chondrogenic phenotype, as prolonged culture of chondrocytes in 21% oxygen has been found to change the phenotype and morphology towards having fibroblastic characteristics (von der Mark et al. 1977).

Improvement of the biological response of implanted chondrocytes can be addressed either by selection of “superior” chondrocytes (Dell'Accio et al. 2001, Saris et al. 2008, Katopodi et al. 2009) or by enhancement of culture strategies. We tried to improve traditional chondrocyte culture conditions by combining 3D and hypoxic culture, and we determined the response-specific gene expression in these different culture environments. We hypothesized that culture in an environment resembling the native one would favor the expression of cartilage-specific genes such as sox9, collagen type 2, and aggrecan.

## Methods

### Cartilage samples

Cartilage biopsies were collected from the intercondylar groove in the distal femur from 12 healthy patients undergoing anterior cruciate ligament reconstruction. The biopsies were collected after obtaining the patients' written consent and the protocol was approved by the local ethics committee under the Danish National Committee on Research Ethics (#M-2008-008). The biopsies were transported to the laboratory in a suspension of DMEM/F-12 with Glutamax (Gibco-Invitrogen), 10% fetal calf serum (FCS) (Invitrogen), streptomycin and penicillin (Sigma-Aldrich), and gentamicin (Sigma-Aldrich). Each biopsy was cut into smaller pieces; this was followed by digestion with 0.1% collagenase II (Gibco) and 0.1% hyaluronidase (Sigma-Aldrich) for 18–20 hours at 37°C in a waterbath. The cells were then washed in DMEM/F-12 (Gibco-Invitrogen) and seeded in a 10-cm^2^ culture dish using standard culture medium containing 10% FCS and the antibiotics mentioned above.

### Scaffolds

Methoxypolyethyleneglycol-block-co-poly(lactide-co-glycolide) (MPEG-PLGA) (50:50 LA:GA) (ASEED; Coloplast A/S, Humlebæk, Denmark) was dissolved in 100 mL 1,4 dioxane overnight at 50°C. 7 mL of polymer solution was poured into a precooled 7.3 × 7.3 cm^2^ aluminum mold and placed in a freeze dryer at –20°C followed by 1 hour at 30°C. The scaffolds were then dried overnight at room temperature in a vacuum dessicator. They were sterilized in 100% ethanol, and dried and packed into aluminized PET bags. The average porosity of the scaffolds was above 90% and the thickness was 2.0 mm. These scaffolds have previously been used in MACI treatment in an animal model (Lind et al. 2008).

### Cell culture

Isolated chondrocytes from each patient were cultured separately in DMEM/F12 medium with antibiotics under normoxia (21% oxygen tension) until they reached confluence. Following trypsinization (with 1.25% trypsin and 5 mM EDTA), the cells were divided to undergo monolayer or scaffold seeding. For monolayer culture, the cells were seeded in 24-well plates at a density of 20,000 cells/cm^2^, i.e. 38,000 cells/well. MPEG-PLGA scaffolds soaked in culture medium (4 mm in diameter) were placed in agarose-coated 24-well plates to prevent the cells from adhering to the wells. 125,000 cells in 10 μL medium were added on top of the wet scaffold (at a seeding concentration of 5 × 10^6^ cells/mL). The scaffolds were left in an incubator for 1.5 h to allow the cells to adhere, followed by gentle addition of 1 mL of culture medium. 24 h after seeding, the cells for baseline investigation (t = 0) were harvested. The remainder was divided into 3 groups for incubation under either normoxia (21% oxygen tension), hypoxia (5% oxygen tension), or severe hypoxia (1% oxygen tension) in a designated hypoxia workstation (Xvivo System, BioSpherix, NY) that was pre-balanced for the desired growth environment. RNA extractions from these subcultures were performed 1, 2, and 6 days after the baseline time point (passage 2). Oxygen and carbon dioxide tensions and also temperature were measured throughout the experiment.

### Extraction of total RNA

Scaffolds with cells were vortexed in 1 mL of TRIzol Reagent (Invitrogen, Taastrup, Denmark) and total RNA was extracted according to the manufacturer's instructions. It was dissolved in nuclease-free water (Ambion, Cambridgeshire, UK). Total RNA from monolayer cells was extracted by means of the GenElute Mammalian Total RNA Miniprep Kit (Sigma-Aldrich). Finally, the RNA concentration was assessed spectrophotometrically by means of the Quant-iT RiboGreen RNA Kit (Molecular Probes) according to the manufacturer's instructions. Each sample was measured in a 96-well plate using a microplate reader. RNA quality analysis was performed using an Agilent 2100 Bioanalyzer (Agilent Technologies, Santa Clara, CA). All measurements were performed in accordance with the manufacturer's instructions.

### qRT-PCR

The RNA samples were treated with DNase I (Ambion) and converted into complementary DNA (cDNA) using the High Capacity cDNA Archive Kit (Applied Biosystems, Naerum, Denmark). Real-time quantitative polymerase chain reaction (qRT-PCR) was performed on a 7500 Fast Real-Time PCR system (Applied Biosystems) using commercially available TaqMan gene expression assays (Applied Biosystems): glyceraldehyde-3-phosphate dehydrogenase (GAPDH; Hs99999905_m1), collagen type 1 alpha 1 (COL1A1; Hs00164004_m1), collagen type 2 alpha 1 (COL2A1; Hs00264051_m1), aggrecan (AGC; Hs00153936_m1), and sox9 (Hs00165814_m1). Standard enzyme and cycling conditions for the 7500 Fast System were used. In order to maximize amplification efficiency, amplicon size for all primer sets was < 180 bp. Cell response to hypoxic challenge was confirmed by means of gene expression analysis of the hypoxia-inducible gene ankyrin repeat domain 37 (ANKRD37) as described by Benita et al. (2009) using TaqMan gene expression assay (Hs00699180_m1; also Applied Biosystems). Values were normalized to those of ribosomal protein L13a (RPL13A; Hs03043885_g1) and β_2_-microglobulin (B2M; Hs99999907_m1), which have proven to be stable under hypoxic conditions (Foldager et al. 2009).

Template cDNA corresponding to 4 ng of RNA was added to each PCR reaction and each biological sample was run in technical duplicates for each gene. Data analysis was performed using 7500 Fast System Sequence Detection software version 1.3 (Applied Biosystems, Naerum, Denmark).

### Live/dead staining

The viability of the cells was checked using live/dead staining (LIVE/DEAD Viability Kit; Molecular Probes). The intracellular esterase activity in live cells converts non-fluorescent calcein AM into highly fluorescent calcein, thus staining live cells green. EthD-1, on the other hand, can only enter cells with damaged membranes. By binding to nucleic acids, a 40-fold enhancement in fluorescence is produced in dead cells and they appear red. The excitation and emission wavelengths for calcein are 494 nm and 517 nm, respectively, and for EthD-1 they are 528 nm and 617 nm. Images were produced using a fluorescence laser microscope (Meta 510; Zeiss Microimaging GmbH, Germany). Z-stacks with 8 and 9 images were used to create 3-dimensional projection images with a depth of 160 μm and 184 μm, respectively.

### Statistics

Relative gene expression levels were log-transformed and residuals were checked for normal distribution using QQ-plots (Stata software version 10.0; StataCorp, College Station, TX). The data were analyzed using repeated-measurements ANOVA taking into account that the cells from each patient were used on both surfaces, at all 3 time points, and in either one or two of the three oxygen tensions. Proc Mixed software (SAS) was used for this analysis. For each of the 3 dependent variables (oxygen tension, surface, time) cells from 6 individual patients were used (n = 6). Regression analyses and stratified regression analyses were carried out in order to detect any correlations between gene expression levels. All p-values less than 0.05 were considered significant.

## Results

**Figure 1.**
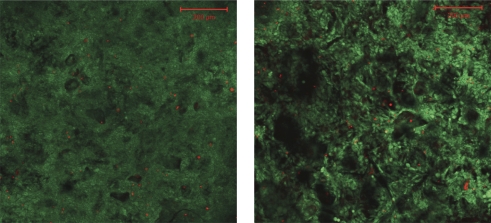
Projection images from confocal microscopy with LIVE/DEAD staining. Viable cells are appear green and dead cells appear red. On day 1 (left panel), the cells were distributed in a layer on the top of the scaffold. On day 3 (right panel), the cells had migrated into the scaffold. The red bars correspond to 200 μm.

**Figure 2.**
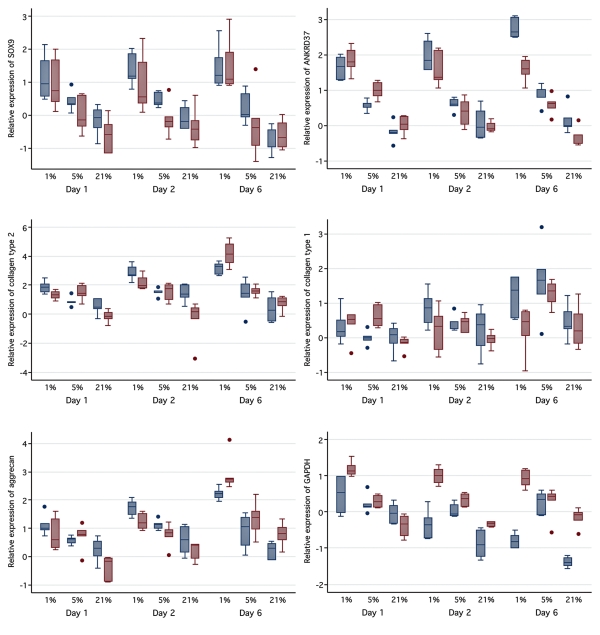
Relative RNA expression levels from qRT-PCR analysis of monolayer cultures (blue) and 3D cultures (red) from 3 different time points and 3 different oxygen tensions. The expression was measured relative to RPL13A and B2M RNA expression. The values are presented as log-transformed in accordance with the statistical method used.

Viability was confirmed by confocal microscopy. On day 1 the cells were spread out on the top of the scaffold, and on day 3 we observed migration of the cells into the scaffold. They were distributed equally in the scaffold and they remained viable throughout the observation period (Figure 1). RNA quality analysis showed RNA integrity values (RINs) of 7.2–9.8 (median 9.0). The algorithm assigns an RIN number score from 1 to 10, where level 10 represents a completely intact RNA and 1 presents a highly degraded RNA. Thus, the RNA quality obtained in this study was sufficient. The relative levels of RNA expression from qRT-PCR analysis are shown in Figure 2.

### Hypoxic challenge

An increase in ANKRD37 RNA expression was observed with lowering of oxygen tension (p < 0.001). No difference in RNA expression over time was observed for the two different culture surfaces. Thus, the hypoxic challenge of the cells was successfully achieved. On day 6, the increase in oxygen tension was dependent on the spatial culture environment, with monolayer culture showing the largest increase in RNA expression (p < 0.001).

### Chondrogenic transcription

With lowering of oxygen tension, an increase in SOX9 RNA expression was observed (p < 0.001). This increase was not dependent on culture time or culture surface. A linear correlation between SOX9 RNA expression and ANKRD37 was observed (R^2^ = 0.59). Thus, the SOX9 expression increased with increase in ANKRD37, the latter corresponding to the hypoxic challenge of the cells.

### Extracellular matrix

Aggrecan RNA expression increased with lowering of oxygen tension at all time points and for both culture surfaces (p < 0.01). In monolayer culture, an increase in aggrecan RNA expression was observed between days 1 and 2 (p < 0.05) but not between these early time points and day 6. However, with 3D culture there was a continuous increase in aggrecan RNA expression between all time points (p < 0.001). Regarding the effect of culture surface on aggrecan RNA expression, the latter was higher in monolayer culture on day 2 (p < 0.01), while on day 6 the expression was higher in 3D culture (p < 0.01). This suggests that there was a positive combined effect of 3D culture and hypoxic conditions on aggrecan RNA expression.

Collagen type 2 RNA expression increased with lowering of oxygen tension at all time points (p < 0.001), but the increase was dependent on both oxygen tension and spatial culture environment. On days 1 and 2, the effect of oxygen on collagen type 2 RNA expression was dependent on the spatial culture environment providing highest expression in monolayer in 21% and 1% (p < 0.05). With severe hypoxia (1%), the expression of collagen type 2 RNA was higher in monolayers on days 1 and 2, while it was higher in 3D culture on day 6 (p < 0.05). The same shift in RNA expression as described above was seen with aggrecan RNA. In 21% oxygen, however, the expression of collagen type 2 RNA was dependent on culture surface in order to increase over time. The highest expression was observed in 3D compared to monolayer (p < 0.01). These findings suggest a regulation of gene expression through both sox9-dependent and sox9-independent pathways.

Collagen type-1 RNA expression was highest in 21% oxygen (p < 0.05), while we observed an interaction between spatial culture environment and culture time with 5% and 1% oxygen. On days 1 and 6 there were interactions between spatial culture environment and oxygen tension (p < 0.05), while the expression of collagen type-1 RNA was dependent on the two individual parameters on day 2 (p < 0.05).

### Cell metabolism

The expression of GAPDH RNA increased with lowering of oxygen tension at all time points (p < 0.001). On both culture surfaces, the effect of low oxygen tension was dependent on the culture time (p < 0.05). In a similar way, the effect of hypoxic culture on GAPDH RNA expression depended on the spatial culture environment (p < 0.001). Stratified regression analyses showed a linear correlation between ANKRD37 RNA expression (i.e. hypoxic response) and GAPDH RNA expression in 3D culture (R^2^ = 0.68) while no correlation was found for monolayer culture (R^2^ < 0.001).

## Discussion

We have found a previously unreported combined effect of hypoxia and 3D culture in porous scaffolds on cartilage-specific gene expression. We found that sox9 RNA expression was only dependent on oxygen tension while collagen type 2 RNA expression was also dependent on the spatial culture environment. The expression of RNA for the other major extracellular component aggrecan was also higher on day 6 in 3D culture than in monolayer culture. Harvested autologous chondrocytes for ACI and chondrocyte-seeded scaffold treatments are usually expanded in vitro in a monolayer culture in ambient air, for subsequent seeding onto a scaffold material 1 or 2 days before implantation. This study highlights some important issues related to this strategy. Our results indicate that an optimized chondrocytic phenotype is achieved by means of hypoxic culture or 3D culture if initiated at least 6 days before cell implantation. In monolayer culture, the expression of aggrecan RNA and collagen type 2 RNA was higher in ambient air in the first 2 days. In order to increase over time, a 3D culture surface would be needed. This suggests that when using chondrocyte-seeded scaffold treatment, early seeding of chondrocytes before implantation might be beneficial for subsequent improvement of cartilage repair.

Our study involved porous MPEG-PLGA polymer scaffolds, as used clinically. Different culture topographies are known to affect cell adhesion patterns, cell orientation, and cell shape (Curtis and Wilkinson 1997). Several studies investigating 3D culture systems have used alginate-based hydrogels (Bonaventure et al. 1994, Gagne et al. 2000). Although alginate is not used clinically, previous studies addressing 3D and hypoxic culture have used this plant-derived material (Murphy 2000a, 2004, Domm 2002). Alginate is derived from plants, and human chondrocytes do not have specific receptors for these materials. This means that the cells are embedded without natural cell-to-matrix interaction, and they cannot therefore receive optimal signals from such plant-derived materials (Rowley et al. 1999). However, these materials are known to both help retain (Hauselmann et al. 1992) and help regain (Benya and Shaffer 1982) the chondrogenic phenotype, and the findings of the present study may therefore not necessarily be related to the spatial topography (as with alginate-based hydrogels) but rather to direct cellular interaction with the porous 3D structure.

The transcription factor sox9 has been shown to directly regulate the expression of collagen type 2 (Bell et al. 1997, Lefebvre et al. 1997). As in previous studies (Murphy 2004, Robins 2005), we have shown that sox9 RNA expression is upregulated under conditions of hypoxia, which suggests that the upregulation of collagen type 2 in this study is a result of an increase in sox9 expression rather than a direct hypoxic response such as the presence of hypoxia response elements (HREs). These elements have not yet been found in the gene encoding collagen type 2. Because of the increase in collagen type 2 expression without a simultaneous increase in sox9 expression, our study suggests that collagen type 2 expression may be regulated by a sox9-independent pathway, and that this pathway may be related to the spatial culture environment. Secondly, sox9 is also known to enhance aggrecan gene promoter/enhancer activity (Sekiya et al. 2000). We found no significant effect of the spatial environment on sox9 expression, although aggrecan expression was higher in 3D culture. After 6 days of culture the expression aggrecan in 3D was higher than in monolayer in all oxygen tensions, although no difference in SOX9 expression between the two spatial environments was found. This suggests a sox9-independent pathway of aggrecan expression.

Coyle et al. (2009) showed that matrix synthesis increased under sustained hypoxia, although the magnitude of the increase in expression of collagen type 2 was independent of culture time. Thus, the increased matrix synthesis was likely to be a result of a continuously high expression of collagen type 2 already on day 1.

GAPDH is an enzyme that mainly serves to break down glucose by catalyzing the sixth step of glycolysis. However, several studies have shown that GAPDH is involved in other cell functions such as phosphotransferase activity (Kim et al. 2002), export of nuclear RNA (Singh and Green 1993), DNA replication and repair (Baxi and Vishwanatha 1995), and activation of gene transcription (Zheng et al. 2003). It is also involved in crucial steps of regulation of gene translocation (Nagy and Rigby 1995). Thus, these findings suggest that GAPDH may not only be a link between the metabolic state and gene transcription; it may also play an important role in inducible cell functions such as protein synthesis. We found that the expression of GAPDH RNA is related to the hypoxic challenge in 3D culture, while no such correlation was found in monolayer culture. This correlation seen in 3D culture may reflect the increased synthesis of extracellular matrix under conditions of hypoxia, but it does not explain the difference between 3D culture and monolayer culture. The large increase in GAPDH expression under hypoxia and the significant differences between monolayer and 3D culture once again emphasizes its inapplicability as a reference gene in RT-PCR analyses (Foldager et al. 2009).

It is known that chondrocytes that are kept for a prolonged period in traditional monolayer culture tend to dedifferentiate. However, several studies have confirmed that chondrocytes can regain their phenotype after a stimulus such as hypoxia. Previous studies have independently shown positive effects of hypoxia (Grimshaw and Mason 2000, 2001, Murphy and Sambanis 2001b, 2004, Malda et al. 2004, Lafont et al. 2008, Coyle et al. 2009), 3D culturing (Hendriks et al. 2006, Takahashi et al. 2007, Barlic et al. 2008, Lin et al. 2009), and a combination of hypoxia and hanging drop culture (Martinez et al. 2008). The hanging drop culture method represents a variant of 3D culture whereby the cells are cultured in media forming droplets, with no cell attachment to the surroundings, which is often used to produce spheroids from embryonic stem cells in vitro (Wang and Yang 2008). This contrasts with our study where porous polymer scaffolds with cell-to-scaffold interaction were used. We found a combined effect of the 2 culture methods, but we were not able to show a decrease in collagen type 1 expression—i.e. reduced dedifferentation. This might be due to the relatively short culture time of 6 days, during which a decrease in sox9 RNA expression that might have been expected in monolayer culture was—likewise—not observed.

One of the limitations of our study is that it only addressed RNA expression and did not investigate quantities of proteins synthesized. Based on such data, only tentative conclusions can be drawn. Also, as topographies and composition of biomaterials vary between different types of scaffolds, this study only reports on the beneficial effect of MPEG-PLGA scaffold culture, although it is likely that other porous 3D polymers exert a similar effect on cartilage-specific gene expression. One should also be aware that the oxygen tension of the surrounding air does not represent the oxygen tension inside the scaffold, as there is an oxygen gradient toward the center of the scaffold. The cells' experience of hypoxic exposure is represented by expression of ANKRD37 RNA (Figure 2). Optimization of chondrocyte populations before implantation has been performed by Saris et al. (2008), by selection of superior cells. However, in vivo investigations comparing early scaffold seeding, hypoxic culture, or the two combined, with conventional culture are yet to be performed. The hypothesis that implanted cells expressing the chondrogenic phenotype would perform better than those showing fibroblastic characteristics would seem obvious. However, the degree of redifferentiation of dedifferentiated chondrocytes after implantation is not well described, but may contribute substantially to sufficient cartilage regeneration. There is a need for animal studies investigating these issues.
